# Supplementary material for: Content-rich biological network constructed by mining PubMed abstracts
Source: BMC Bioinformatics. 2004 Oct 8;5:147. doi: 10.1186/1471-2105-5-147 (PMC528731; doi:10.1186/1471-2105-5-147)
Supplement: Additional File 2 — The original results of the above study (non-essential files are deleted to keep the file size under the limit set by BMC bioinformatics). [file 1471-2105-5-147-S2.bz2 › chilibotAdditionalFile2/dip10/492ID7706250E10655/html/TGFB1_TGFBR1.html]

 


 **TGFB1** and **TGFBR1** 
  
Found 188 abstracts in PubMed, retrieved 10.  
 

 What does Google say? 
 PDF only 
| .edu only 

---

**Interactive relationship** (e.g. stimulation, inhibition, etc)

**Non-interactive relationship** (e.g. studied together, co-existance, homology, etc.)

- These data demonstrate that an autocrine transforming growth factor beta1  [ **TGFB1** ]  ALK5  [ **TGFBR1** ]  pathway is a negative feedback mechanism for epidermal growth factor induced normal human keratinocyte growth.  Ref: 12787131 J Invest Dermatol, 2003
- TGF beta1  [ **TGFB1** ] , ALK 1, ALK 5  [ **TGFBR1** ] , and TbetaRII were also detected by immunohistochemistry.  Ref: 12883979 Pediatr NephrolPediatr Nephrol, 2003
- These results indicate that ACE inhibition in the developing kidney decreases TGF beta1  [ **TGFB1** ] , ALK 1, and ALK 5  [ **TGFBR1** ]  expression, which may account for renal growth impairment.  Ref: 12883979 Pediatr NephrolPediatr Nephrol, 2003
- Newborn rat pups were treated with enalapril 30 mg kg per day or vehicle for 7 days, and kidneys were removed for Western blotting of TGF beta1  [ **TGFB1** ] , ALK 1, ALK 5  [ **TGFBR1** ] , and TbetaRII, and for RT PCR of ALK 5  [ **TGFBR1** ]  and TbetaRII.  Ref: 12883979 Pediatr NephrolPediatr Nephrol, 2003
- This study was designed to investigate the relationship between renal growth impairment and the expression of transforming growth factor beta1  [ **TGFB1** ]  TGF beta1  [ **TGFB1** ] , TGF beta  [ **TGFB1** ]  receptor I TbetaRI, activin like kinase ALK 1 and ALK 5  [ **TGFBR1** ] , and TGF beta  [ **TGFB1** ]  receptor II TbetaRII.  Ref: 12883979 Pediatr NephrolPediatr Nephrol, 2003
- Enalapril decreased renal TGF beta1  [ **TGFB1** ] , ALK 1, and ALK 5  [ **TGFBR1** ]  protein expression P< ; 0.05.  Ref: 12883979 Pediatr NephrolPediatr Nephrol, 2003
